# Supplementary material for: Sub-diffraction error mapping for localisation microscopy images
Source: Nat Commun. 2021 Sep 23;12:5611. doi: 10.1038/s41467-021-25812-z (PMC8460687; doi:10.1038/s41467-021-25812-z)
Supplement: Supplementary file 8 — Description of Additional Supplementary Files [file 41467_2021_25812_MOESM8_ESM.pdf]

Title: Supplementary Software:
Description: The HAWKMAN software is provided as an ImageJ plugin

Title: Supplementary Movie 1:
Description: Full results of HAWKMAN analysis of the Localization Microscopy Challenge microtubule data using Single-Emitter fitting in ThunderSTORM. Top to bottom show the sharpening map, the structure map and the confidence map respectively. Each frame corresponds to a 20nm increase in the length scale used, ranging from 20nm (frame 1) to 300nm (frame15). Colours and scale are as indicated in Fig.1 of the main text. For clarity of display one level of dilation has been applied to the structure maps.

Title: Supplementary Movie 2:
Description: Full results of HAWKMAN analysis of the Localization Microscopy Challenge microtubule data using Multi-Emitter fitting in ThunderSTORM. Colours, maps, frames and scale are as Supplementary Video 1. For clarity of display one level of dilation has been applied to the structure maps.

Title: Supplementary Movie 3:
Description: Full results of HAWKMAN analysis of the Localization Microscopy Challenge microtubule data using the SRRF algorithm with default parameters (see methods). Colours, maps, frames and scale are as Supplementary Video 1. For clarity of display one level of dilation has been applied to the structure maps.

Title: Supplementary Movie 4:
Description: Full results of HAWKMAN analysis of the Localization Microscopy Challenge microtubule data using the SRRF algorithm with optimised parameters (see methods). Colours, maps, frames and scale are as Supplementary Video 1. For clarity of display one level of dilation has been applied to the structure maps.

Title: Supplementary Movie 5:
Description: Full results of HAWKMAN analysis of the Localization Microscopy Challenge microtubule data using 4th order SOFI. Colours, maps, frames and scale are as Supplementary Video 1. For clarity of display one level of dilation has been applied to the structure maps.
