# Supplementary material for: Sub-diffraction error mapping for localisation microscopy images
Source: Nat Commun. 2021 Sep 23;12:5611. doi: 10.1038/s41467-021-25812-z (PMC8460687; doi:10.1038/s41467-021-25812-z)
Supplement: Supplementary file 10 — Reporting Summary [file 41467_2021_25812_MOESM10_ESM.pdf]

nature research

Corresponding author(s): Susan Cox

Last updated by author(s): Mar 11, 2021

Reporting Summary

Nature Research wishes to improve the reproducibility of the work that we publish. This form provides structure for consistency and transparency in reporting. For further information on Nature Research policies, see our Editorial Policies and the Editorial Policy Checklist.

Statistics

For all statistical analyses, confirm that the following items are present in the figure legend, table legend, main text, or Methods section.

n/a	Confirmed	
x		The exact sample size (n) for each experimental group/condition, given as a discrete number and unit of measurement
	x	A statement on whether measurements were taken from distinct samples or whether the same sample was measured repeatedly
x		The statistical test(s) used AND whether they are one- or two-sided<br>*Only common tests should be described solely by name; describe more complex techniques in the Methods section.*
x		A description of all covariates tested
x		A description of any assumptions or corrections, such as tests of normality and adjustment for multiple comparisons
x		A full description of the statistical parameters including central tendency (e.g. means) or other basic estimates (e.g. regression coefficient) AND variation (e.g. standard deviation) or associated estimates of uncertainty (e.g. confidence intervals)
x		For null hypothesis testing, the test statistic (e.g. F, t, r) with confidence intervals, effect sizes, degrees of freedom and P value noted<br>*Give P values as exact values whenever suitable.*
x		For Bayesian analysis, information on the choice of priors and Markov chain Monte Carlo settings
x		For hierarchical and complex designs, identification of the appropriate level for tests and full reporting of outcomes
x		Estimates of effect sizes (e.g. Cohen's d, Pearson's r), indicating how they were calculated

Our web collection on statistics for biologists contains articles on many of the points above.

Software and code

Policy information about availability of computer code

Data collection	NIkon Elements v5.2 and MicroManager v1.3 were used to control the microscope
Data analysis	ImageJ v1.52, ThunderSTORM v1.3, SRRFv1.14, SQUIRRELv1.0, balancedSOFI v1.0, DeconSTORM v1.0 CSSTORM v1.0, HAWK v1.1<br>All software is publicly available free of charge, published and referenced in manuscript.

For manuscripts utilizing custom algorithms or software that are central to the research but not yet described in published literature, software must be made available to editors and reviewers. We strongly encourage code deposition in a community repository (e.g. GitHub). See the Nature Research guidelines for submitting code & software for further information.

Data

Policy information about availability of data

All manuscripts must include a data availability statement. This statement should provide the following information, where applicable:

- Accession codes, unique identifiers, or web links for publicly available datasets
- A list of figures that have associated raw data
- A description of any restrictions on data availability

The raw image sequences and reconstructions of the data supporting this research will be downloadable from the Kings College London Server on publication. Raw data from the Localization Microscopy Challenge is publicly available from http://bigwww.epfl.ch/smlm/

Field-specific reporting

Please select the one below that is the best fit for your research. If you are not sure, read the appropriate sections before making your selection.

☒ Life sciences ☐ Behavioural & social sciences ☐ Ecological, evolutionary & environmental sciences

For a reference copy of the document with all sections, see nature.com/documents/nr-reporting-summary-flat.pdf

Life sciences study design

All studies must disclose on these points even when the disclosure is negative.

Sample size	No sampling of population data is relevant to this work. Comparison of analysis is made on individual datasets where prior knowledge of the expected result exists.
Data exclusions	No data where excluded. Analysis is performed on individual datasets
Replication	The research consists of a computer program applied to individual datasets or simulations. It contains no random or stochastic elements and running the analysis will produced identical results. It has been tested on both Windows and Linux systems
Randomization	No group allocation of data was performed in the research, randomization is therefor not relevant.
Blinding	No group allocation of data was performed in the research, blinding is therefor not relevant.

Reporting for specific materials, systems and methods

We require information from authors about some types of materials, experimental systems and methods used in many studies. Here, indicate whether each material, system or method listed is relevant to your study. If you are not sure if a list item applies to your research, read the appropriate section before selecting a response.

Materials & experimental systems

n/a	Involved in the study	
☐	☒	Antibodies
☐	☒	Eukaryotic cell lines
☒	☐	Palaeontology and archaeology
☒	☐	Animals and other organisms
☒	☐	Human research participants
☒	☐	Clinical data
☒	☐	Dual use research of concern

Methods

n/a	Involved in the study	
☒	☐	ChIP-seq
☒	☐	Flow cytometry
☒	☐	MRI-based neuroimaging

Antibodies

Antibodies used	Monoclonal Anti-Beta-Tubulin antibody produced in mouse, Sigma-Aldrich T8328 ; Anti-Clathrin heavy chain antibody, abcam ab21679 ; Goat anti-Mouse IgG (H+L) Cross-Adsorbws Secondary Antibody Alexa Fluor 647, Invitrogen A-21235 ; Goat anti-Rabit IgG (H+L) Cross-Adsorbed Secondary Antibody Alexa Fluor 647, Invitrogen A-21244 ; Tom20 Antibody FL-145) rabit polyclonal IgG , Santa Cruze Biotechnology, sc-11415 ; Goat anti-Rabbit IgG (H+L) Highly Cross-Adsorbed Secondary Antibody Alexa Flour 647, Molecular Probes A-21245. Rabbit polyclonal antibody Z1Z2, Young, P. EMBO J., 1998 PMID 9501083 provided by M. Gautel, KCL. Goat anti-rabbit secondary-AlexaFluor 647 Life Technologies A-21244.
Validation	All antibodies used in this study have been validated. For specific validation information visit manufacturer websites for all antibodies except Z1Z2.<br>Beta Tubulin T8328 - https://www.sigmaaldrich.com/catalog/product/sigma/t8328?lang=en&region=GB<br>Clathrin ab21679 - https://www.abcam.com/clathrin-heavy-chain-antibody-ab21679.html<br>Goat-anti-mouse-AF647 (thermofisher) A-21235 - https://www.thermofisher.com/antibody/product/Goat-anti-Mouse-IgG-H-L-Cross-Adsorbed-Secondary-Antibody-Polyclonal/A-21235<br>Goat-anti-rabit-AF647 (invitrogen) A-21244 - https://www.thermofisher.com/antibody/product/Goat-anti-Rabbit-IgG-H-L-Cross-AdsorbedSecondary-Antibody-Polyclonal/A-21244<br>Tom20 sc-11415 - https://www.scbt.com/p/tom20-antibody-fl-145<br>Goat-anti-Rabit-AF647 (Molecular probes) A-21245 - https://www.thermofisher.com/antibody/product/Goat-anti-Rabbit-IgG-H-L-Highly-Cross-Adsorbed-Secondary-Antibody-Polyclonal/A-21245<br>Goat-anti-Rabit-AF647 (life technologies) A-21244 - https://www.thermofisher.com/antibody/product/Goat-anti-Rabbit-IgG-H-L-Cross-Adsorbed-Secondary-Antibody-Polyclonal/A-21244<br>For Z1Z2 antibody see Young, P. EMBO J. 1998 Vol.17 No.8 pp1614-1624.<br>The antibody labeling used in this research is solely for the purpose of producing representative structures for STORM imaging. The

analysis and results presented do not depend on the specificity of any antibody.

Eukaryotic cell lines

Policy information about cell lines

Cell line source(s)	COS-7 cells (CRL-1651,ATCC) , HeLa (ATCC)
Authentication	COS-7 Cell line authenticated by supplier, no further authentication was performed. HeLa were not tested for authenticity
Mycoplasma contamination	HeLa - The cells are regularly tested for mycoplasma infection and any that test positive are destroyed. The Cos-7 cells tested negative for mycroplasma infection
Commonly misidentified lines (See ICLAC register)	No commonly misidentified cell lines were used.
